# Dimensions of Child Maltreatment in Australians With a History of Out-of-Home Care

**DOI:** 10.1177/10775595241297944

**Published:** 2024-11-05

**Authors:** Lottie G. Harris, Daryl J. Higgins, Megan L. Willis, David Lawrence, Franziska Meinck, Hannah J. Thomas, Eva Malacova, James G. Scott, Rosana Pacella, Divna M. Haslam

**Affiliations:** 1 Faculty of Education and Arts, Institute of Child Protection Studies, 95359 Australian Catholic University, Melbourne, VIC, Australia; 2 Institute of Child Protection Studies, 95359 Australian Catholic University, Melbourne, VIC, Australia; 3 Faculty of Health Sciences, School of Behavioural and Health Sciences, 95359 Australian Catholic University, Sydney, NSW, Australia; 4 School of Population Health, 1649 Curtin University, Perth, WA, Australia; 5 School of Social & Political Science, University of Edinburgh, Edinburgh, UK; 6 Faculty of Humanities, North-West University, Vanderbijlpark, South Africa; 7 School of Public Health, University of the Witwatersrand, Johannesburg, South Africa; 8 Queensland Centre for Mental Health Research, Wacol, QLD, Australia; 9 Faculty of Medicine, 541177 The University of Queensland, Brisbane, QLD, Australia; 10 QIMR Berghofer, Medical Research Institute, Brisbane, QLD, Australia; 11 Child Health Research Centre, 541177 The University of Queensland, Brisbane, QLD, Australia; 12 Child and Youth Mental Health Service, Children’s Health Qld, South Brisbane, QLD, Australia; 13 Institute for Lifecourse Development, 4918 University of Greenwich, London, UK; 14 School of Law, 1969 Queensland University of Technology, Brisbane, QLD, Australia; 15 Parenting and Family Support Centre, University of Queensland, Brisbane, QLD, Australia

**Keywords:** foster care, child protective services, maltreatment, measurement, public health, t-tests

## Abstract

Research suggests that the dimensions of childhood maltreatment (type, age of onset, duration, frequency and perpetrator) play an important role in determining health and wellbeing outcomes, though little information is available on these dimensions for any care experienced cohorts. This study aimed to determine if any variation in maltreatment dimensions were experienced between two subsets of the nationally representative Australian Child Maltreatment Study, both of which reported childhood maltreatment histories: care-experienced (*n* = 358) and non-care-experienced (*n* = 4922). Using a series of independent t-tests and chi-square tests, we compared the two groups on seven dimensions (number of maltreatment types, range of maltreatment items, age of onset, duration, frequency, perpetrator number, and perpetrator type) for the five child maltreatment types (physical, emotional, sexual abuse, neglect, and exposure to domestic violence). Results showed that the care-experienced group reported a higher intensity of maltreatment, being younger when maltreatment first started, experiencing greater variety of maltreatment types, for longer periods, more times and by more perpetrators than maltreated people with no care experience. We conclude that children and young people in out-of-home care experience maltreatment at a higher intensity than the rest of the population, which has implications for effective treatment.

## Introduction

Practitioners and researchers are increasingly aware of the impact that early childhood adversity—particularly child maltreatment—has on both immediate and long-term health and development outcomes (John et al., 2019; Winter et al., 2022). Historically, researchers have measured maltreatment based on exposure to discrete maltreatment types focusing particularly on physical abuse and sexual abuse (Jackson et al., 2019). As the field has developed, however, the nuances of child maltreatment are being explored with increasing sophistication (Gabrielli & Jackson, 2019). Well-established evidence as to the high prevalence and substantial impact of cumulative maltreatment experiences has shown that systems of measuring childhood maltreatment by type alone do not fully account for the heterogeneity of such experiences nor the breadth of their outcomes (Cicchetti & Toth, 2005; Hazzard et al., 2019). More recently, researchers have recommended various dimensional models of child maltreatment as valuable alternatives (McLaughlin et al., 2021; Russotti et al., 2021). Maltreatment dimensions commonly include type, severity, frequency, age or developmental stage at onset, duration, and relationship to perpetrator. These characteristics of maltreatment exposure (hereon referred to as ‘dimensions’) have been theorised to account for variation in a range of outcomes, yet the literature has been slow to consistently integrate these concepts into population level studies on the epidemiology of child maltreatment (Davis et al., 2019).

Examining the maltreatment experiences of the general population has important benefits at the macro and micro levels (English, 2003). Applying a public health approach to the prevention of child maltreatment requires both secondary and tertiary prevention efforts to target the right people at the right level with the right resources. To mitigate the effects of maltreatment, adults providing services to children (e.g., carers, case workers, educators, and health professionals, etc.) require a sound knowledge and keen awareness of the prevalence and intensity of maltreatment in particular cohorts. In this study we aim to determine whether there are variations in the experiences of childhood maltreatment to subsequently provide data that can be used to make decisions on whether a particular group, in this case the care-experienced cohort, would benefit from targeted and/or prioritized interventions. Specifically, determining whether the OOHC cohort experiences a different level of maltreatment intensity has implications for treatment planning and resource allocation across all child-servicing sectors.

Our previous study explored the maltreatment experience of participants who did and did not report an OOHC experience and showed differential mental health outcomes between the two groups, even when matched on the number of maltreatment types experienced (Harris et al., 2024). These findings began our investigation into the possible causes of differential outcomes for care- and non-care-experienced cohorts, hypothesising that maltreatment intensity may be a key factor.

The term ‘care-experienced’ is widely used in academic and day-to-day language in the UK as an umbrella term when referring to a person who is either currently or has previously been cared for by the state. This term is less common in Australia; however, it is becoming increasingly popular in academic writing internationally, given its broad scope and preference by people with lived experience of state care (Pinkney & Walker, 2020). We adopted the use of the term ‘care-experienced’ for our study for this and other practical reasons, as detailed in the methodology section.

### Differences Between Maltreatment Types

Maltreatment type is the most common dimension of maltreatment exposure examined within the literature (Jackson et al., 2019). Data availability is likely a key factor in why this dimension has received more attention than others as studies typically differentiate between maltreatment experiences based on type. Despite vast methodological and conceptual differences on maltreatment types, these tend to include physical abuse, sexual abuse, emotional abuse, neglect, and more recently, exposure to domestic violence (Mathews et al., 2023). Maltreatment type has long been hypothesized to be a key dimension in understanding outcome variation. A comprehensive review by Noll (2021) concluded that child sexual abuse presents a unique risk factor for anxiety, mood and substance use disorders even when accounting for other maltreatment types. This aligns with a systematic review that assessed the differential effects of maltreatment type on critical brain structures and demonstrated preliminary evidence that both sexual and emotional abuse (but not physical abuse or neglect) affect brain volume and activity in unique ways (Cassiers et al., 2018). Even within maltreatment types, there are important nuances. Grummitt et al. (2022) demonstrated that emotional neglect was a more significant predictor of depression and anxiety than physical neglect in a sample of young Australian adults.

### Multi-Type Maltreatment

Exposure to two or more childhood maltreatment types, termed ‘multi-type maltreatment’ is typically a more common experience than only having ever experienced one type (Higgins et al., 2023; Warmingham et al., 2019). Previous analysis using the Australian Child Maltreatment Study data showed that the care-experienced group reported severe multi-type maltreatment at a higher rate (18.5% experienced all five types) than their non-care-experienced peers (2.6% experienced all five types) and any multi-type maltreatment was the most common maltreatment pattern for the care-experienced group – experienced by 79.0% (cf 37.1% of non-care-experienced group *([redacted for anonymous review])*. Experiencing multi-type maltreatment has also been repeatedly shown to result in poorer physical and mental health outcomes (Bijlsma et al., 2023; Clemens et al., 2018; Huguenel et al., 2021; Scott et al., 2023).

### Age of Onset

The age of maltreatment onset as a maltreatment dimension is relevant given literature within the fields of neurobiology and developmental psychology on sensitive periods of brain development (Larsen & Luna, 2018; Nelson & Gabard-Durnam, 2020). Many studies have tested the hypothesis that the earlier in life maltreatment occurred, the greater the severity of symptoms and poorer outcomes experienced. Kaplow and Widom (2007) found that the earlier age of onset of physical abuse, sexual abuse and neglect predicted more symptoms of psychological distress later in life.

Despite the conceptual rationale and numerous empirical research, the evidence remains challenging to interpret. In a systematic review of 118 studies, Schaefer et al. (2022) found the research on whether there are any periods of increased vulnerability at which maltreatment experience would cause *significantly* poorer psychological outcomes was inconclusive. However, these results were likely related to methodological diversity, the low quality of studies, and the multicollinearity of other maltreatment dimensions.

### Duration

Duration represents the total time of exposure to maltreatment, a term often used interchangeably with chronicity. Cowell et al. (2015) used the term chronicity to represent the number of developmental periods that maltreatment spanned in their sample of 370 children. The children who experienced maltreatment over the course of three or more developmental periods performed significantly worse on a series of inhibitory control, memory, and motor tasks than both the non-maltreated control group as well as those who experienced maltreatment over one or two developmental periods. They found little difference in test scores between the children who experienced maltreatment in one developmental period and those who experienced maltreatment over two developmental periods suggesting a certain amount of exposure has limited effect up until a point (three developmental periods). In a separate review of the literature on the neurobiological, cognitive, and psychiatric impacts of child maltreatment, abuse duration was associated with lower brain volume, executive dysfunction and other cognitive impairments (e.g., lower IQ), as well as deficits in memory (Carvalho et al., 2016).

### Frequency

Frequency of maltreatment is typically operationalised as a total count of incidences of a certain experience however some studies have dichotomised frequency into ‘occurred one time’ versus ‘occurred more than one time’ (for example, Van Wert et al., 2017). Much like other dimensions, many have argued that prolonged or repeated experience is likely to produce different outcomes than single time events (Jackson et al., 2014). McGuire et al. (2018) for example reported that sexual abuse frequency significantly influenced externalising behaviours. Similarly, Lantos et al. (2019) reported maltreatment frequency as a moderator of offending behaviour where increased maltreatment frequency was associated with greater violent and non-violent offending in adolescence.

### Perpetrators of Maltreatment

Historically, maltreatment perpetrator characteristics---such as age, gender, and relationship to child--have been understudied and often overlooked in favour of the more common maltreatment dimensions previously mentioned. A US longitudinal research study with youth in foster care examined maltreatment perpetrator characteristics (type of relationship to victim, frequency of maltreatment, perpetrator by maltreatment type; Bennett et al., 2023). They found that the constellations of perpetrators for physical and psychological abuse were very similar in that biological parents and peers were most often reported as perpetrators. In comparison, community members and peers were more likely than caregivers to be reported as perpetrators of sexual abuse. Bennett et al. illuminated the differing patterns of perpetration as an important characteristic of maltreatment. In addition, two recent Australian studies concluded that a history of child maltreatment where the perpetrator was either a mother or father resulted in poorer adult wellbeing scores than participants whose maltreatment perpetrator was another adult (over 18 years old), but not a parent (Jankovic et al., 2022, 2024).

Despite growing interest in dimensional models of maltreatment, significant gaps and inconsistencies within the literature persist that obfuscate the true effect that these exposure characteristics may have on outcomes. A key limitation in the current evidence is that studies rarely examine all maltreatment dimensions together and are therefore at risk of overestimating the effect of an individual dimension. The landscape of the current literature demonstrates great variation in the impact of dimensions largely because of the heterogeneity of maltreatment experiences making it challenging to synthesise and draw accurate conclusions. To continue to build our understanding of such experiences, it is imperative to include all possible dimensions in a combined analytical model.

There is also potential for practical application of a dimensional model of maltreatment. In Australia, no consistent national data are available on the dimensions of maltreatment experienced by children in care, beyond the individual types – and even when multiple types are reported, details are often only provided for the type that caused the most harm (Australian Institute of Health and Welfare [AIHW], 2023). Practitioners across child support systems have limited access to accurate data on the lifetime maltreatment experiences of the children and families they are working with. By understanding the typical dimensions of maltreatment experienced by those in and out of the care system, this paper will help inform policy and practice and equip practitioners with population level data that can help inform their history taking.

## The Present Study

Our primary aim is to examine whether care-experienced people and non-care-experienced people report different dimensions to their maltreatment experiences. We hypothesise that the maltreatment of the care-experienced group was of a quantifiably higher intensity (i.e., maltreatment involved a combination of more maltreatment types, started at an earlier age, lasted longer, happened more frequently, and was perpetrated by more people) than those who reported maltreatment but no experience of OOHC. We use the term ‘maltreatment intensity’ throughout this study to refer to overall experience of maltreatment as other common terms such as ‘chronic’ or ‘severe’ maltreatment have specific meanings in the literature that do not encompass the breadth of the experience that we focus on. It is well established that children and young people who enter OOHC are highly likely to have experienced maltreatment, however the way maltreatment is experienced is also likely to be substantially different for this cohort. We expect that exploring these patterns in detail will support two goals. Firstly, for policy makers and practitioners, these data will allow for a more fine-tuned public health response to the prevention and treatment of child maltreatment across Australia. Secondly, we propose that by exploring the effect of individual maltreatment dimensions we can examine, which, if any should be included in future comprehensive models aimed at quantifying the intensity of childhood maltreatment. Using a multidimensional approach, we aim to provide a more nuanced measurement and analysis strategy to disentangling the complex interactions associated with child maltreatment.

## Methodology

We used existing data from the Australian Child Maltreatment Study (ACMS). The ACMS used a cross-sectional design to conduct computer-assisted telephone interviews with a random sample of 8503 Australians aged 16 years and older between April and October 2021. The survey asked participants to retrospectively recount their childhood experiences of maltreatment among other childhood and adulthood experiences. The Juvenile Victimisation Questionnaire (JVQ) – R2: Adapted Version (ACMS) was used to capture the self-reported experiences of child maltreatment from this sample (Mathews et al., 2023). For this study, we analysed a subgroup of the ACMS: comprising 5280 participants who reported ever having experienced childhood maltreatment, which constituted 62.2% of the whole sample (Higgins et al., 2023). Further methodological details of the ACMS are reported in another publication (Haslam et al., 2023).

### Out-of-Home Care

The ACMS included a single question about alternative care experiences. Regardless of whether they endorsed any of the child maltreatment questions, all participants were asked, “Were you ever placed in out-of-home care, such as foster care or kinship care?” with responses recorded as: yes, no, don’t know, or refuse. Consistent with previous studies (*[redacted for anonymous review]*), we adopted a conservative approach: all participants who answered, ‘Yes’ to this question were classified as the ‘care-experienced group’. All participants who answered ‘No,’ or ‘I don’t know’, or refused to answer were categorised into the non-care-experienced group. For those in the care-experienced group we cannot be clear on the timing of entry to care nor the timing of the maltreatment they experienced in relation to time spent in OOHC. As such this study is unable to and does not claim causation, that differential maltreatment influenced entry into OOHC, rather we describe the different experiences of two groups.

Participants of the ACMS were aged 16+, which meant that there was a possibility for our sample to include people (aged 16–21) who were in OOHC at the time of the study as well as people who had been in OOHC at some point during their childhood before engaging in the study. Given the range of time periods in which participants may have been in OOHC, we believe that the term ‘care-experienced’ best represents our sample in its entirety. Similarly, the term ‘non-care-experienced’, best describes our control group, which includes people aged 16+ who never spent any time in OOHC. The ACMS surveyed a representative sample of the Australian general population; however, it should be noted that the two groups compared in this study are only representative of the population who reported ever experiencing any child maltreatment. For the sake of brevity, we use the terms ‘care-experienced’ and ‘non-care experienced’ throughout this study.

### Maltreatment Dimensions

#### Maltreatment Type and Items

Five maltreatment types were assessed in the ACMS: physical abuse, emotional abuse, sexual abuse, neglect, and exposure to domestic violence. These five maltreatment types were rigorously tested and validated prior to the ACMS survey instrument going live, leaning on robust conceptual and practical evidence to inform each maltreatment construct (Haslam et al., 2023). For each maltreatment type participants were asked whether they had ever experienced any of the associated items. The ACMS maltreatment items are phrased as specific behaviours that fall under the category of each maltreatment type. As an example, physical abuse was comprised of two items, (1) “Did an adult ever hit, punch, kick, or physically hurt you?” and (2) “Did an adult ever beat you up, hit you on the head or face, choke you, or burn you?” Participants were classified as having experienced a given maltreatment type (i.e., physical abuse) if they endorsed any associated item (beat up, vs. hit/punch/kick). Categorisation is described in detail in Haslam et al. (2023) though in brief, physical abuse was measured by two items, neglect and emotional abuse by three items and, sexual abuse, and exposure to domestic violence by four items each. To be categorised as having experienced physical abuse, sexual abuse or exposure to domestic violence, a participant need only have endorsed one maltreatment type item one time. If any of the neglect or emotional abuse items were endorsed, participants were asked “did this happen over a period of days, weeks, months, or years?” Participants who responded only with ‘days’ were not categorised as having experienced emotional abuse or neglect given both maltreatment types conceptually require exposure to have occurred over a prolonged period. Only participants who reported either weeks, months or years were categorised as having experienced the associated maltreatment type. Affirmative responses to all items of each maltreatment type triggered follow up questions which gathered greater detail on additional dimensions including the age of onset, frequency, duration and perpetrator details.

#### Age of Onset

Age was measured for all maltreatment items by asking ACMS participants to report the earliest age that each maltreatment item occurred. Ages ranged from zero (before participants first birthday) to 17.

#### Duration

The duration in years for which maltreatment occurred was calculated as earliest age of cessation minus earliest age of onset for each maltreatment item (e.g., age 14 years minus age 5 years = 9 years duration). It should be noted that the term chronicity has often been used in place of the word ‘duration’ as we have described it, however in other studies it has been used to mean the number of maltreatment experiences, which we call ‘frequency’. To ensure clarity in this study we use the term duration rather than chronicity.

#### Frequency

Frequency of incidents of physical and sexual abuse, and exposure to domestic violence were measured by asking participants to recount to the best of their ability, the number of times each maltreatment item occurred. Responses were originally reported as discrete numeric variables ranging from one time to more than one hundred times. Frequency as a dimension was not assessed for either neglect or emotional abuse as these two maltreatment types conceptually do not occur as single incidences.

#### Perpetrator

Perpetration was assessed by asking participants the question: “Who were all the people who did this to you?” after endorsement of each maltreatment item for physical, sexual and emotional abuse. Respondents could answer using the language they found most appropriate and could list as many perpetrators as necessary for each item. Interviewers coded responses into a list of up to 42 perpetrator categories such as ‘male relative who did not live with you’, and ‘stepmother, or father’s live-in girlfriend’. It is worthwhile to note here that the term perpetrator has often been associated with criminal intent and individual accountability. Discussion in the child welfare and domestic violence fields have challenged this assumption particularly regarding peers and parents whose actions are better explained as a response to sociopolitical pressures than a deliberate intention to harm (Children and Young Peoples Centre for Justice, 2023). In the context of this study the term perpetrator is used for the sake of consistency with other studies using this dataset including the original ACMS questionnaire.

### Data Analysis

All analyses in this study compared those who reported having ever experienced any maltreatment by care-experienced (*n* = 358) and non-care experienced (*n* = 4922) participant groups. We completed the full analysis of this study twice: once completely excluding those participants who reported ‘I don’t know’ or refused in response to the question regarding alternative care. Another time we included these participants into the non-care-experienced group. This was to determine whether the inclusion of those who were unsure or didn’t wish to answer the OOHC question significantly altered any results. We found no significant differences between the two analytical approaches, therefore, in an effort to include as much data as possible, we included those participants who reported ‘No’, ‘I don’t know’ and those who refused the question into the non-care-experienced group.

**Figure 1.**
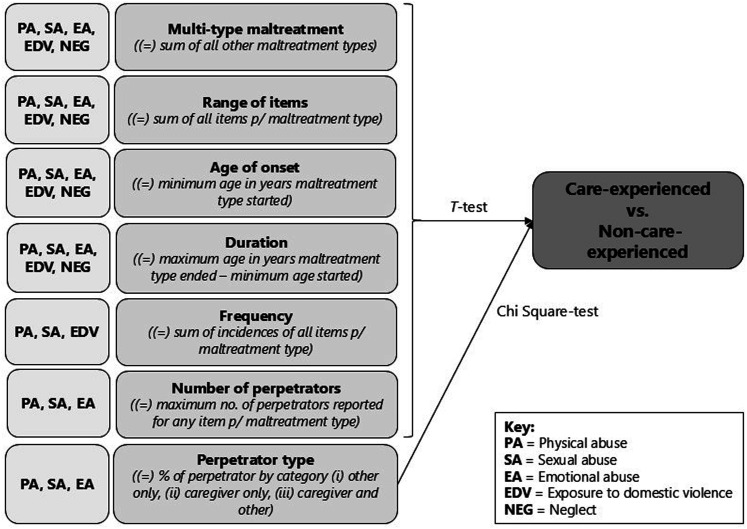
Visual representation of the analysis plan for this study.

Based on the literature and available data, we tested seven dimensions individually for each of the five maltreatment types. The seven dimensions of interest in this study are: number of maltreatment types (multi-type maltreatment), range of maltreatment items, age of maltreatment type onset, duration of maltreatment type, frequency of maltreatment type, perpetrator number and perpetrator type. To simplify our analysis model, for all dimensions except for ‘range of items’, we combined maltreatment items to create one variable per dimension per maltreatment type. Ideally other dimensions such as age at entry to care, and location/setting of maltreatment would have been included in our model however, we were restricted by the variables collected as part of the wider Australian Child Maltreatment Study. The analytical methods used in this study for deriving each dimension variable are described below and presented visually in Figure 1.

#### Multi-Type Maltreatment

The prevalence of multi-type maltreatment has been previously reported for this sample (Higgins et al., 2023), therefore we knew a-priori that a significant proportion of the maltreated cohort had experienced more than one maltreatment type. We calculated the maximum number of other maltreatment types experienced for each participant. For example, if a participant endorsed physical abuse, we calculated if and how many other maltreatment types (of emotional abuse, sexual abuse, neglect, exposure to domestic violence) they had also endorsed giving a multi-type maltreatment score. Participants may not have endorsed any other maltreatment type therefore the range of scores for this dimension was 0–4.

#### Range of Maltreatment Items

The sum of the number of items within each maltreatment type endorsed created our range of items dimension. For example, the neglect maltreatment type included three items: environmental neglect, nutritional and physical neglect and medical neglect. A participant who reported experiencing two of the three neglect items was coded as ‘2’ on our neglect range of items variable, while another participant who reported experiencing one of the three items was coded as ‘1’ on the same scale. We calculated item scores for each participant per maltreatment type where the maximum score was dependent on the number of items allocated to each maltreatment type. The item range was 1–2 for physical abuse, 1–3 for emotional abuse and neglect and 1–4 for sexual abuse and exposure to domestic violence. This method was considered conceptually sound as a proxy for severity as research has repeatedly shown that the greater variety of maltreatment experiences the poorer the outcomes.

#### Age of Onset

We calculated age of onset for each maltreatment type by identifying the earliest reported age of onset for any of the items for that maltreatment type. Thus, if a respondent endorsed both physical abuse items, where one began at four years and the other at seven years, the age of onset for physical abuse would be four years. This approach was repeated for each maltreatment type.

#### Duration

The duration of each maltreatment item per type was calculated in years by subtracting the age of onset from the age of cessation for each item. We selected the item that lasted the greatest number of years to represent the duration dimension for each maltreatment type. For example, if a participant reported an experience of both threats of violence and physical violence (two items of the exposure to domestic violence maltreatment type) where the threats lasted a total of four years and the physical violence lasted three years, the participants exposure to domestic violence duration score would be four years.

#### Frequency

The frequency of physical and sexual abuse and exposure to domestic violence was calculated as the total sum of maltreatment incidences that participants reported across each of the endorsed items. For example, if a participant reported that abusive exposure or voyeurism (one of the sexual abuse items) occurred 10 times and contact abuse (another sexual abuse item) occurred 15 times, the total frequency count for sexual abuse for that participant would be 25.

#### Perpetrator Number

Participants reported the number of perpetrators for each endorsed maltreatment item. The number of perpetrators was calculated by coding the highest number of perpetrators reported across *all* maltreatment items per type. For example, if a participant reported three perpetrators were responsible for a given physical abuse item but five people were responsible for perpetrating a different physical abuse item, the highest perpetrator number would be allocated, in this case five. Creating a variable that represents the highest number reported for a single item (rather than summing the number of all perpetrators) was considered the best approach conceptually as we predicted that the perpetrators of one maltreatment item would likely be the same as the perpetrators of other items and hoped to avoid conflating the actual number by not counting the same perpetrator twice.

#### Perpetrator Type

Noting that participants could have experienced maltreatment from multiple perpetrators within each child maltreatment type, we created a variable for perpetrator type by combining perpetrators into a nominal variable with three groups: ‘non-caregiver only,’ ‘caregiver only’, and ‘caregiver and other’. The caregiver group included biological parents, live-in stepparents, and foster, kinship or guardianship carers as these all provided direct care in a parenting type role warranting inclusion in the caregiver category. We categorised all other perpetrator types as ‘non-caregiver’ which included groups such as ‘male or female relative that lived in the home’, ‘parent’s boyfriend or girlfriend who did not live in the home’ and ‘male or female schoolteacher, sports coach or religious leader.’ Given that children and young people enter OOHC most often because of parent or caregiver-perpetrated maltreatment, it was important to develop a method of distinguishing between ‘perpetrator as caregiver’ and ‘perpetrator as other’. Participants who did not provide a perpetrator for each maltreatment item endorsed were excluded from this part of the analysis though included in other tests. Although the concept of child maltreatment is often associated with the caregiver as perpetrator, in this study we apply the World Health Organization ([WHO], 2006, p. 9) definition which states that maltreatment occurs in the “context of a relationship of responsibility, trust or power.”

### Testing Group Differences

To determine whether there were any statistically significant differences in the maltreatment experiences of the care-experienced and non-care-experienced groups, a series of independent samples *t*-tests were run. We used *t-*tests as the dimension variables were mostly continuous (number of types, range of items, age of onset, duration, frequency and number of perpetrators). We used a chi-square test for the only categorical dimension in our model which was perpetrator type. We conducted independent *t*-tests and chi-square tests for each dimension per maltreatment type, resulting in 29 individual tests. Alpha was set at *p* < .05 for all analyses. Given that not all maltreatment items were endorsed by each respondent, we were unable to compare every dimension across maltreatment type in a single analysis model (i.e., analysis of variance) nor did we consider that adjusting for multiple comparisons appropriate or informative as per arguments made by Perneger (1998). Our intention in this study was primarily to determine whether the dimensions of maltreatment are important factors in understanding maltreatment intensity to lay the foundations for future research, which may incorporate these dimensions into a single model. To reduce further complexity in the analysis, we did not include any covariates beyond the dimension variables already outlined.

## Results

### Multi-Type Maltreatment

As shown in Table 1, for all five maltreatment types, there was a statistically significant difference between the care- and non-care-experienced groups on the average number of other maltreatment types they also experienced. The care-experienced group consistently reported experiencing more maltreatment types than the non-care-experienced group.

**Table 1.** Weighted Means (With 95% CI) and T-test Results of Maltreatment Dimensions (Age of Onset, Range, Duration, Frequency, and Number of Perpetrators), by Experience of Type of Child Maltreatment.

		Mean (CI)		*t*-test		
		Maltreated and care-experienced (*n* = 358)	Maltreated and non-care-experienced (*n* = 4922)	t value	df	*p* value
Physical abuse	*n*	261	2362			
	Multi-type maltreatment (0–4)	2.8 (2.7–3)	2.2 (2.1–2.2)	8.6	2256	<.002
	Range of items *(1-2)*	1.4 (1.3–1.4)	1.2 (1.2–1.2)	4.6	2622	<.001
	Age of onset *(years)*	7.3 (6.7–7.9)	8.5 (8.3–8.7)	3.5	2618	<.001
	Duration *(years)*	7.1 (6.5–7.8)	5.4 (5.2–5.6)	4.8	2614	<.001
	Frequency	45 (34.4–55.8)	24 (21.7–26.1)	3.8	3704	<.001
	Number of perpetrator types	1.6 (1.5–1.7)	1.5 (1.5–1.5)	1.2	2465	.242
Sexual abuse	*n*	201	2147			
	Multi-type maltreatment *(0-4)*	3 (2.9–3.2)	2.2 (2.1–2.2)	11.6	3486	<.001
	Range of items *(1-4)*	2.6 (2.4–2.8)	2 (2–2.1)	5.2	2347	<.001
	Age of onset *(years)*	9 (8.3–9.7)	10.4 (10.2–10.6)	3.7	2342	<.001
	Duration *(years)*	4 (3.3–4.6)	2.3 (2.2–2.5)	5	2341	<.001
	Frequency	48 (33.6–62.9)	22 (19.2–25.3)	3.4	2293	<.001
	Number of perpetrator types	1.5 (1.4–1.7)	1.2 (1.2–1.3)	4.3	2280	<.001
Emotional abuse	*n*	278	2465			
	Multi-type maltreatment *(0-4)*	2.8 (2.7–2.9)	2.2 (2.1–2.2)	8.5	2507	<.000
	Range of items *(1-3)*	2.2 (2.1–2.3)	1.8 (1.7–1.8)	7.6	2742	<.001
	Age of onset *(years)*	5.7 (5.2–6.2)	7.9 (7.7–8.1)	7.9	2714	<.001
	Duration *(years)*	9.8 (9.2–10.4)	8.2 (7.9–8.4)	5.1	2714	<.001
	Number of perpetrator types	1.7 (1.6–1.8)	1.6 (1.5–1.6)	2.5	2674	.014
Exposure to domestic violence	*n*	288	3199			
	Multi-type maltreatment *(0-4)*	2.8 (2.6–2.9)	2.1 (2.0–2.1)	9.3	2731	<.000
	Range of items *(1-4)*	2.9 (2.7–3.1)	2.1 (2–2.1)	8.9	3486	<.001
	Age of onset *(years)*	6.3 (5.8–6.8)	8.2 (8–8.4)	6.9	3464	<.001
	Duration *(years)*	7.5 (6.8–8.1)	5.9 (5.7–6.1)	4.6	3459	<.001
	Frequency	117.1 (98.3–135.9)	53.3 (49.3–57.4)	6.5	3378	<.001
Neglect	*n*	150	609			
	Multi-type maltreatment *(0-4)*	2.9 (2.7–3)	2.1 (2–2.1)	11.3	5251	<.001
	Range of items *(1-3)*	1.7 (1.5–1.8)	1.3 (1.3–1.4)	4.1	876	<.001
	Age of onset *(years)*	6.9 (6.2–7.7)	7.8 (7.5–8.2)	2.1	867	.032
	Duration *(years)*	7.4 (6.5–8.2)	6.8 (6.4–7.3)	2	867	.322

### Range of Maltreatment Items

Across all maltreatment types, the care-experienced group reported experiencing significantly more items compared to the non-care-experienced group (Table 1).

### Age of Onset

Similarly, the *t*-test results for age of onset revealed that all maltreatment types resulted in statistically significant differences between the care-experienced and non-care-experienced groups (Table 1). The care-experienced group reported earlier ages of onset for all five maltreatment types. The greatest age difference was shown for emotional abuse where the earliest mean age of onset for the care-experienced group was 5.7 years old whereas the mean age for the non-care-experienced group was 8.2 years.

### Duration

Results showed that the duration of maltreatment was statistically significantly higher for the care-experienced than the non-care-experienced for physical abuse, sexual abuse, emotional abuse, and exposure to domestic violence (Table 1). Emotional abuse was the maltreatment type experienced for the longest time in years for both groups though again, the care experienced group experienced emotional abuse for longer. There was no statistically significant difference between groups on the duration of neglect.

### Frequency

Those who reported an out-of-home care experience compared to those who never experienced out-of-home care reported physical abuse, sexual abuse and exposure to domestic violence to have occurred significantly more frequently, see Table 1. For sexual abuse and exposure to domestic violence, the frequency was over double the number for the care-experienced group compared to the non-care-experienced group.

### Number of Perpetrator Types

The care-experienced group reported sexual abuse and emotional abuse by a greater number of perpetrator types compared to those in the non-care-experienced group (Table 1). While the care-experienced group reported more perpetrator types of physical abuse, than the non-care-experienced group, this difference was non-significant.

### Perpetrator Type

Finally, the chi-square test results (Table 2) showed that there was a statistically significant difference in the proportion of the care and non-care-experienced groups who reported each perpetrator type for sexual and emotional abuse, but not physical abuse. Participants without a care experience were more likely to report that a caregiver was the only perpetrator of emotional abuse compared to the care-experienced group whereas the care experienced group were more likely to report that caregivers as well as others were the perpetrators of emotional abuse. For both physical and emotional abuse most of both groups reported maltreatment having been perpetrated by their caregiver only, whereas for sexual abuse, the highest proportion of perpetrators were non-caregivers. This was true for both the care and non-care-experienced groups though a much higher proportion of the care-experienced group reported sexual abuse perpetration by ‘caregivers and other’ than the non-care-experienced group.

**Table 2.** Weighted Prevalence Estimates (With 95% CI) and Chi-Square Results of Maltreatment Dimensions (Perpetrator Type), by Experience of Type of Child Maltreatment.

		*n*, %, (95% CI)		Chi-square test		
		Maltreated and care-experienced (*n* = 358)	Maltreated and non-care-experienced (*n* = 4922)	Pearsons chi-square	df	*p* value
Physical abuse	*n* ^a^	261	2362			
	Other only	23, 12.2% (7.7%–18.7%)	205, 10.2% (8.7%–11.9%)	8.3	2	.094
	Caregiver only	165, 68.5% (60.9%–75.2%)	1717, 76.2% (73.9%–78.4%)			
	Caregiver & other	54, 19.3% (14%–26%)	298, 13.6% (11.9%–15.6%)			
Sexual abuse	*n* ^a^	201	2147			
	Other only	151, 79.8% (72.5%–85.6%)	1946, 92.4% (90.8%–93.7%)	83.8	2	<0.001
	Caregiver only	9, 4.6% (2.1%–9.4%)	88, 4.7% (3.7%–6.1%)			
	Caregiver & other	30, 15.6% (10.6%–22.5%)	57, 2.9% (2.1%–4%)			
Emotional abuse	*n* ^a^	278	2465			
	Other only	8, 3.8% (1.5%–9.2%)	32, 1.7% (1%–2.6%)	16.5	1.9	.026
	Caregiver only	225, 81.3% (74.8%–86.5%)	2189, 88.7% (86.6%–90.2%)			
	Caregiver & other	45, 14.9% (10.5%–20.7%)	244, 9.7% (8.3%–11.3%)			

^a^Some participants did not provide a perpetrator for a maltreatment type they endorsed therefore the *n* for perpetrators does not equal the total number of people who reported each maltreatment type.

Overall, we conducted 29 tests across seven dimensions for the five maltreatment types. All but three tests produced results that showed a statistically significant difference, at the *p* < .05 level, between the care- and non-care-experienced groups. The large number of significant differences that all present in a consistent direction (i.e., the care-experienced group reporting a higher level of maltreatment intensity than the non-care-experienced group) demonstrate a strong likelihood that these results and pattern of maltreatment did not occur by chance.

## Discussion

This study aimed to determine whether the care and non-care-experienced maltreatment groups of the ACMS sample who reported any maltreated, experienced substantially different maltreatment histories and whether exploring maltreatment dimensions would be a beneficial way to determine whether differences exist. Taken together, the results revealed that those with a care experience were consistently more likely to experience multi-type maltreatment, a greater range of maltreatment items, maltreatment starting at an earlier age, lasting for a longer period, and perpetrated by more people than the non-care-experienced group. The consistency of the results illustrates the strength of this pattern, that the care-experienced group experience maltreatment differently, and at a greater intensity than other maltreated people who did not have a care experience. Taking in the big picture, these results suggest that the threshold for entry to care in Australia, is having experienced maltreatment at a high level of intensity. This corresponds to policy initiatives that describe placement in out-of-home care as the intervention of last resort (AIHW 2021). Our findings broadly align with those reported by Biehal et al. (2018) who showed that in their sample of 380 children, the ‘ever in care’ group were more likely to attain a maltreatment rating of ‘high severity’ compared with those in the ‘never in care’ group.

At the macro level, identifying distinct groups of people whose maltreatment experience is objectively more intense is useful in ensuring that targeted support for healing and recovery reach the groups most in need. On each dimension that we assessed, those with a care experience reported increased maltreatment intensity than their non-care-experienced but maltreated peers, with all but a couple being statistically significant. These results and our novel methodology present a series of important findings and implications relevant to policy makers, practitioners, and researchers.

### Maltreatment Experiences Associated With a Greater Number of Maltreatment Types and Range of Items

Results for those who reported sexual abuse in our study are similar to those of Vachon et al. (2015) who claim that sexual abuse (without any other co-occurring child maltreatment type) is rare, only 1% of their study sample (2292 US children aged 5–13) and are not representative of the most common experience of child maltreatment. Our study did show generally that experiencing a single type of maltreatment, of any type was not typical for the care-experienced group. Findings also showed that maltreatment items are highly correlated; where there is an experience of one item, there is likely to be more. For the care experienced group, the range of items was greater than the non-care-experienced group illustrating that their experience of maltreatment is vast and varied. It is likely that families who come to the attention of child welfare services do so because they are challenged by myriad issues that influence risk factors for child maltreatment. Multiple risk factors such as intergenerational trauma, substance use, and mental ill health, when combined are likely to lead to the enactment of multiple maltreatment experiences. It is assumed that families that face fewer challenges leading to less varied and pronounced maltreatment are similarly less likely to be involved with child welfare and out-of-home care systems.

The implications of the high prevalence of multi-type maltreatment (particularly severe multi-type maltreatment) in the care-experienced group are two-fold. First, for researchers, this study adds to the growing body of literature that urge for models of maltreatment measurement to account for complex interactions between maltreatment types. Second, in practice, this data shows the widespread experience of multi-type maltreatment in children and young people who experience OOHC and as such, practitioners would expect such experiences to be the basis for therapeutic intervention even when the exact data is limited.

### Early Onset Maltreatment

For each type of maltreatment, care-experienced participants reported that the maltreatment started at a *younger* age compared to the non-care-experienced group. Comparable data specific to children in OOHC is limited, though this finding is corroborated by national statistics that report the rates of admission into OOHC are highest for children under one year of age in Australia (AIHW, 2022). Green et al. (2018) demonstrated that a younger age (under 5) at which maltreatment was experienced increased physical and mental health vulnerabilities. Our data combined with this growing evidence suggests that intervention is required significantly earlier in children’s lives than is currently occurring and that intervention must be effective for younger ages. Increasing targeted supports to families identified as exhibiting risk factors for maltreatment, should come earlier in the lives of children, even prenatally for some families. Early childhood education and parenting programs can provide positive avenues to engage families in prevention efforts early (Holzer et al., 2006; Prinz et al., 2009). Although our data could not provide information on the timing of entry to care for participants, earlier entry to OOHC has been shown to reduce potential health and educational difficulties than for children who enter later in childhood experience may face (Hu et al., 2024). There is a strong argument for earlier engagement with intensive support systems for children who are at significant risk of maltreatment at a young age, though OOHC should continue to be considered only as a maltreatment intervention when all others are unavailable or unsuccessful.

### Maltreatment of Greater Duration

Maltreatment experienced by those with a care experience lasted longer than their non-care-experienced peers, regardless of the maltreatment type with all but neglect resulting in statistically significant results. For the care-experienced group, emotional abuse lasted on average more than nine years while physical abuse, neglect and exposure to domestic violence all lasted on average more than seven years. These findings suggest that intervention is not appropriately targeted to risks and/or is initiated as efficiently and/or as effectively it could be, thereby prolonging exposure and further elevating risk of poor health outcomes. This may possibly be the result of the earlier age of maltreatment onset in this group combined with later in life detection. Alternatively, we may consider that the children who enter OOHC are likely from families whose psycho-social challenges are chronic and multi-causal and therefore require significant coordinated investment to change. Parents whose children were not removed may have had fewer challenges in changing their behaviour towards less harmful patterns. It is noteworthy that emotional abuse was reported to have been experienced for the longest duration. This is particularly concerning as data shows emotional abuse contributes to socio-emotional problems in children (Cecil et al., 2017; Gama et al., 2021). It is also known to be an incredibly difficult maltreatment type to identify (compared with more visible signs of physical abuse or neglect) and to substantiate in child protection investigations (North, 2019). This may result in the emotional abuse not being addressed in as timely a way as more overt maltreatment types for the care-experienced group.

### Maltreatment of Greater Frequency

The care-experienced group reported that each maltreatment type occurred a substantial number of times. This finding is not surprising given the care experienced group’s maltreatment experiences started earlier and lasted for longer than the non-care-experienced group. For this group, the maltreatment occurred for longer periods on average, therefore it follows that this likely equates to more individual incidences of maltreatment types. As we have hypothesised for other maltreatment dimensions, it may be that families of participants who had not spent any time in OOHC experienced shorter periods of family dysfunction and/or experienced less chronic or entrenched issues which they found easier to address not warranting OOHC intervention However, the impact of repeated maltreatment experiences cannot be understated. Li and Godinet (2014) showed that for children with repeated notifications of maltreatment from age 0–12, the impact of increased maltreatment frequency was evident in their internalising and externalising behaviours and became much more pronounced as they grew older. Based on our understanding of the neurobiological impact of chronic stress, experiencing repeated instances of maltreatment is known to cause maladaptation in children’s threat response and emotional regulation systems, interfere with their sense of safety and core attachment relationships (Cross et al., 2017; Warmingham et al., 2023; Wright & Folger, 2017).

### Perpetration by Both Caregivers and Others

The rates at which the three perpetrator groups (other only, caregiver only, and both caregiver and other) were reported as inflicting emotional and sexual abuse were significantly different between those who experienced OOHC and those who did not. Only a very small percentage of the care-experienced group (2.8%, 0.9% and 2.1%) reported that the person who perpetrated the physical, sexual and emotional abuse was a ‘foster, kinship or guardianship carer.’ Most participants reported perpetrators in the caregiver category as biological parents or stepparents. Caregivers made up most of the emotional abuse perpetrators for both the care-and non-care experienced groups, which is unsurprising given the nature of emotional abuse. This implies that caregivers should be the main target for any parenting support that addresses emotional abuse specifically.

For sexual abuse, the proportions of perpetrator type were markedly different to other maltreatment types. People other than parents or caregivers where the perpetrator type most often reported for sexual abuse by both care-and non-care-experienced people, aligning with other research on sexual abuse perpetrator type (Ferragut et al., 2021; Finkelhor et al., 2014). However, the care-experienced group reported that both a caregiver and a non-caregiver perpetrated sexual abuse more than five times more often than the non-care-experienced group (15.6% vs. 2.9%). These results firstly suggest that children and young people in OOHC have a much higher rate of caregiver perpetrated sexual abuse than others, which has implications for how family arrangements should be assessed and supported by statutory child protection bodies. In addition, such results show that this group is particularly vulnerable to sexual abuse by multiple people which reflects the research that identifies co-occurring maltreatment types as a key risk factor for sexual abuse (Scoglio et al., 2021).

Beyond findings on specific maltreatment dimensions, the overarching conclusion is that care-experienced people are likely to have experienced a greater number of types of maltreatment, for longer periods, more times and by more types of people than those who were maltreated but who did not enter OOHC. From one perspective the findings suggest the child protection system is doing what it is intended to do by enacting the most intrusive of interventions by the state (i.e., removal from parental care and placement into alternative care) for those who are unable to remain safely in the care of their parent(s). In this regard we would expect that children who enter OOHC would have experienced the most intense maltreatment. On the other hand, our data show that despite intervention in the form of OOHC, the maltreatment this group experienced was extreme. Ideally, such intense experiences of maltreatment need to be addressed early, quickly, and use the most effective evidence-based strategies. The OOHC group will likely need specialist and consistent therapeutic supports to adequately address the impacts of their maltreatment experiences.

In lieu of detailed data on the specific characteristics of the maltreatment experiences of individual children and young people currently in OOHC in Australia, the findings of this study should be used as a general reminder of the significant abuse and neglect that they are likely to have endured. In addition, this is an important reminder for all professionals working with children and young people in OOHC, including child protection case workers, teachers, health professionals, youth justice workers and Carers, to approach current behavioural and developmental presentations through an informed understanding of their maltreatment history.

## Limitations and Future Directions

Despite efforts to develop the evidence for the adoption of a complex and nuanced dimensional model, this study encountered some limitations. First, the study design involved participant recall as the sole source of data. Although first person retrospective accounts of child maltreatment have been shown to be more reflective of actual experience (Cho & Jackson, 2016), the ACMS survey relied on participants to know and remember each of their maltreatment experiences. This is likely to be extremely difficult for maltreatment experiences that were particularly frequent, chronic or those which happened at an early age. Similarly, it is unlikely that participants kept track of discrete incidents during childhood in a manner that allowed them to note exactly how many times or for how long an experience occurred. This places limitations on the level of detail able to be captured about maltreatment experiences in a survey such as the ACMS. The accuracy of self-reported retrospective maltreatment data to this level of specificity should therefore be taken with caution and considered an estimation as close as is possible Equally, accounts of OOHC experiences rely on participants identifying their experiences as such, which may be challenging, for example, for people who spent most of their childhood in the care of a close relative (i.e., kinship care) but who may not consider themselves as being in OOHC. Some participants were unsure how to respond to this question and given our conservative approach to categorizing care experience, we may have excluded several participants who should be included in the OOHC group. Future studies using a similar method to analyze maltreatment dimensions may benefit from including data from multiple sources to elevate the accuracy of the maltreatment information.

Second, and critically, we did not know the timing of the experience of maltreatment and whether it occurred before, during or after their OOHC experience. Additionally, the data does not inform us on whether the maltreatment continued or commenced while they were in an OOHC placement. The ACMS questionnaire included only one question regarding OOHC experience, which limited our ability to definitively comment on the order of care and maltreatment experiences. It could be the case that a participant experienced a stable and therapeutic period of their childhood while in OOHC but then moved to another placement or were reunified with family where they were exposed to maltreatment. The exact order of events is unknown, and therefore the known impact of OOHC alone is limited.

Third, due to the relatively small sample of the care experienced group, we were unable to do any age-specific or gender-specific analyses. Therefore, we could not test if this ‘higher intensity for care-experienced participants’ observation is consistent across different age groups, or whether the maltreatment experiences of younger participants with a care experience differ from the older age groups. Further exploration with larger cohorts would be a necessary next step in confirming such patterns.

Finally, we did not include any other potentially important covariates in our analysis, such as participant gender, family socioeconomic status during childhood, or exposure to adverse childhood experiences (other than child maltreatment). Partially this was due to our particular focus exploring the dimensions of maltreatment as covariates themselves, but also due to limitations in the data available. Further models incorporating dimensions of maltreatment should consider which other variables may play an important role in the effects of child maltreatment experiences.

## Conclusion

The current study clearly showed that for every maltreatment type, on almost every dimension, there were important differences between groups. The care-experienced group reported a much higher intensity of maltreatment overall. Our perspective is that participants who reported less intense maltreatment are likely to have grown up in families with fewer risk factors which made them less likely to come to the attention of child protection services. On the other hand, the care-experienced group are likely to have been in OOHC by virtue of their intense level of maltreatment experience; both because such a level of maltreatment is more easily identifiable by statutory authorities and because families that engage in intense maltreatment are likely to have more entrenched challenges that are harder to resolve quickly and effectively.

This information is likely to be helpful, not only to child welfare professionals but other adults in a child’s life such as their teachers, nurses, mentors etc. These results that show the high likelihood that the care-experienced cohort have suffered intensive maltreatment, should increase the impetus to apply trauma—informed practice across all institutions that care-experienced child encounter. The results demonstrate the utility of incorporating seven unique maltreatment dimensions into any future maltreatment analysis model. Few child maltreatment research methodologies have taken steps towards increasing the analysis complexity despite calls for such action. The heterogenous nature of child maltreatment requires a measurement model that can account for such complexity so that the nuances of experience can be understood thoroughly and to ensure that critical differences are not overlooked. To the best of our knowledge, the present model is the first to successfully incorporate this breadth of maltreatment dimensions and has demonstrated utility in helping shape our understanding of the experiences of a care-experienced group beyond what has been previously possible. Our model accounts in part for both developmental and ecological theories of child maltreatment by incorporating contextual information such as what, how, when and by whom into our analysis of childhood maltreatment experience.
